# IL-24 contributes to skin inflammation in Para-Phenylenediamine-induced contact hypersensitivity

**DOI:** 10.1038/s41598-018-38156-4

**Published:** 2019-02-12

**Authors:** Astrid B. Van Belle, Perrine M. Cochez, Magali de Heusch, Lisa Pointner, Remi Opsomer, Peggy Raynaud, Younes Achouri, Emilie Hendrickx, Pamela Cheou, Guy Warnier, Jean-Christophe Renauld, Marie Baeck, Laure Dumoutier

**Affiliations:** 1 de Duve Institute, Université catholique de Louvain, Brussels, Belgium; 2 Institut de Neurosciences, Université catholique de Louvain, Brussels, Belgium; 3 Ludwig Institute for Cancer Research, Brussels branch, Brussels, Belgium; 4 Department of Dermatology, Cliniques Universitaires Saint-Luc, UCL, B-1200 Brussels, Belgium; 5 Institut de Recherche Expérimentale et Clinique, Université catholique de Louvain, Brussels, Belgium

## Abstract

Para-Phenylenediamine (PPD) is an aromatic amine used in hair dyes and in temporary black henna tattoos, which is a frequent cause of allergic contact dermatitis (ACD). ACD is a skin inflammatory reaction characterized by modifications such as spongiosis, exocytosis and acanthosis. The aim of this study is to characterize the expression and the role of IL-20-related cytokines, including IL-19, IL-20, IL-22 and IL-24, in ACD. The expression of *IL19*, *IL20*, *IL22* and *IL24* is increased in affected skin from PPD allergic patients compared with uninvolved skin. In addition, the expression of these cytokines positively correlates with clinical symptoms. To assess their role in ACD, we set up a mouse model of PPD-induced allergic contact dermatitis and we showed that, in contrast to *Il22*-deficient mice, *Il22ra1*-, *Il20rb*- and *Il24*-deficient mice are partially protected against development of PPD-induced contact hypersensitivity. These mice have decreased ear thickening and less acanthosis compared with WT mice after PPD treatment. In addition, the absence of IL-22R, IL-20R2 or IL-24 affects the recruitment of neutrophils into the skin but not the total IgE production. Taken together, these results demonstrate the implication of IL-24 via the IL-20R type II receptor in the inflammatory process of ACD.

## Introduction

Para-Phenylenediamine (PPD) is an aromatic amine used in hair dyes and in temporary black henna tattoos^1^. Because of its potent allergenic properties, hairdressers or consumers of hair dye products can develop allergic contact dermatitis (ACD) and the overall prevalence of PPD contact allergy is 0.8% in Europe’s population^1–4^.

The clinical symptoms of PPD-induced ACD after hair dyeing are erythema, massive edema, vesicles and oozing of the face, the scalp and the neck. The diagnosis is obtained by applying patch tests containing the suspected substance on the skin of allergic patients. At histological level, the main features of ACD are spongiosis (edema), exocytosis (infiltration of immune cells in the epidermis) and an important inflammatory infiltrate in the dermis. A slight acanthosis (thickening of the epidermis) is also observed and is accentuated in chronic ACD^5^. At the cellular and molecular level, it is well known that both cytokines and T lymphocytes are important in the mediation of the allergic response^6^. A prerequisite for an antigen-specific response in ACD is the sensitization phase during which T cells are primed by the hapten-carrier complex. Subsequent contact with the same allergen will induce strong T cell responses and recruitment of these hapten-specific T lymphocytes in the skin, which, in turn, will lead to the epidermal changes described above^6^. The allergic response might be Th1-, Th2-, or Th17-dominant, depending on several factors such as the kind of hapten, the genetics of individuals, the site of contact, the state of skin before the contact (inflamed or not) and the microbiota^7^.

Beside T cell-derived cytokines, pro-inflammatory cytokines such as IL-1β, IL-18 and TNFα are important during both sensitization and elicitation phases of ACD^7–9^. The current main treatment against ACD is to avoid the contact with the allergen and associated molecules. As reactions are frequently severe, topical or systemic treatment by corticosteroid is necessary^10^. A better understanding of PPD allergy mechanisms seems to be essential to improve prevention and treatment.

Because IL-20-related cytokines are known to play an important role in skin inflammatory diseases such as psoriasis^11^, they could be actors in the ACD reaction. IL-20-related cytokines are produced by immune cells such as monocytes and T lymphocytes and are involved in the maintenance of the epidermal barrier integrity by promoting antimicrobial peptide production, chemokine expression and keratinocyte proliferation^11^. These cytokines play redundant roles because they share common receptor complexes. IL-19, IL-20 and IL-24 can bind to the “type I IL-20 receptor” composed of IL-20R1 and IL-20R2. The “type II IL-20 receptor” consists of IL-22R and IL-20R2 and binds IL-20 and IL-24^12^. Finally, IL-22 signals through a complex composed of an IL-22R subunit and IL-10R2.

Even if the biological activities of IL-20-related cytokines are beneficial during wound healing or pathogen invasion, these cytokines might play a detrimental role in inflammatory skin disorders^11^. For instance, they are upregulated in skin lesions from psoriatic patients^13–15^ and we showed that IL-22 is implicated in keratinocyte proliferation and abnormal differentiation as well as in neutrophil infiltration in a mouse model of psoriasis^16^. In addition, transgenic mice for IL-20, IL-22 and IL-24 but not IL-19 display a thickened skin due to acanthosis, demonstrating the role of IL-22R-binding cytokines in this skin inflammatory disorder^17–19^. In ACD, very little is known about the role of IL-20-related cytokines. IL-22 is found in the serum of nickel-allergic patients^20^ and is produced by CD4^+^ T lymphocytes that are present in the skin of these patients^21,22^. An increase of *Il22* expression in the skin has also been reported in murine contact hypersensitivity (CHS) models induced by TNCB or oxazolone^23,24^. In addition, the expression of *Il19* and *Il24* is also increased in a mouse model of CHS induced by DNFB^14^. In another model of TNCB-induced CHS, it was shown that *Il20rb*-deficient mice were more affected than WT mice, suggesting that IL-19, IL-20 and IL-24 play a protective role^25^.

In PPD-induced contact dermatitis, no information is available about the expression of IL-20-related cytokines in the skin of patients, nor in animal models. Here, we show that the expression of *IL19*, *IL22* and *IL24* was increased in both human and mouse models of PPD-induced CHS. In addition, *Il22ra1*-, *Il20rb*- and *Il24*-deficient mice were partially protected against development of CHS, demonstrating a detrimental role of IL-24 via its effect on type II IL-20R in PPD-induced CHS.

## Results

### IL-20 subfamily cytokines are expressed in the skin of PPD-allergic patients

**Figure 1:**
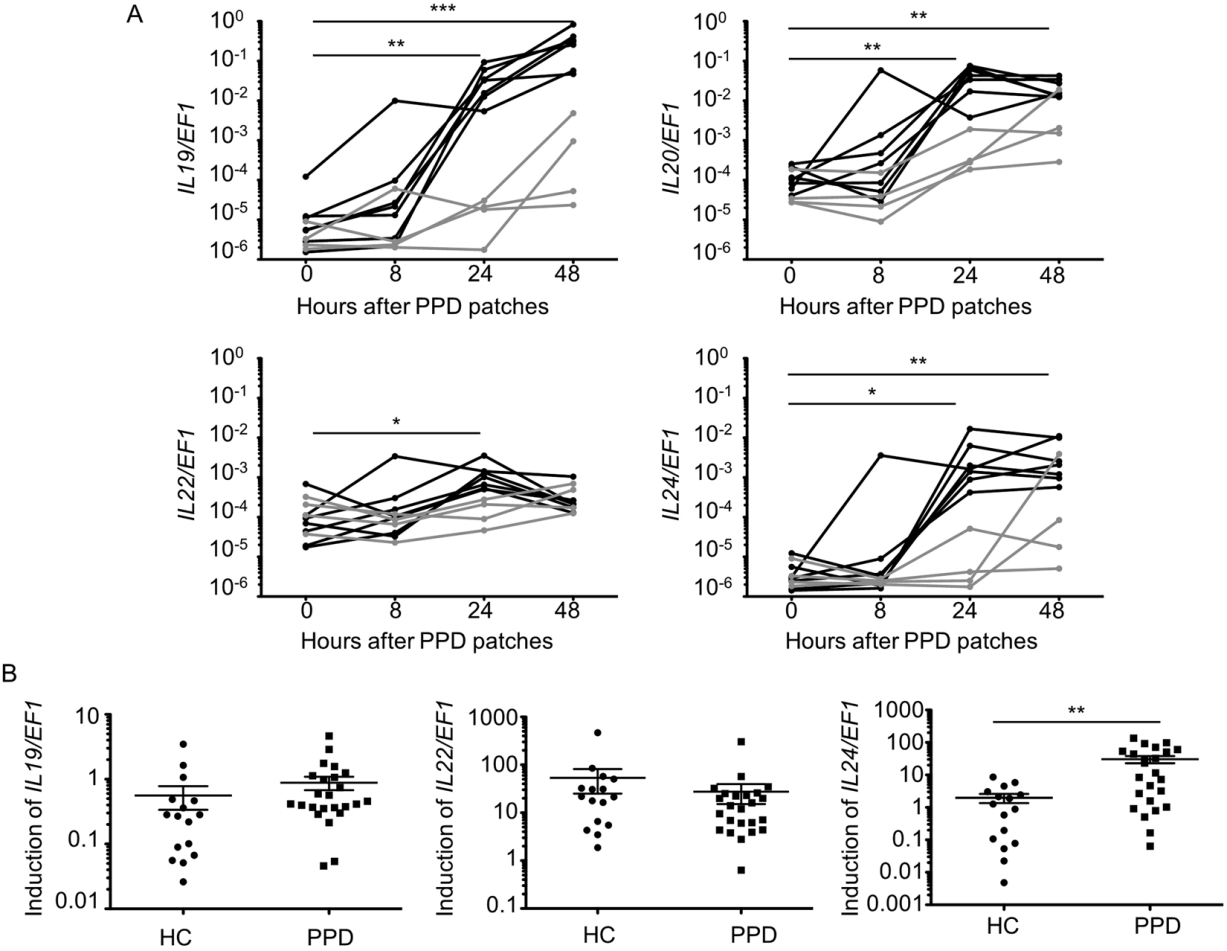
Expression of IL-20-related cytokines is increased in skin of PPD-allergic patients. (**A**) RNA was isolated from healthy skin and patch test biopsies (at indicated period of time) of allergic patients (N = 11). B. RNA was isolated from PBMCs of healthy control (HC, N = 16) and PPD-allergic patients (PPD, N = 24). PBMCs were stimulated with anti-CD3, anti-CD28 and PPD for 48 hours. Next, qPCR for *IL19*, *IL20*, *IL22*, *IL24* and *EF1* mRNA expression were performed. (**A**) Black curves represent patients with at least a positive patch test reaction at 24 hours. Grey curves represent patients with a negative patch test reaction at 24 hours. (**B**) The induction is calculated by comparing the expression of cytokines in stimulated PBMCs vs unstimulated PBMCs. ^*^p < 0.05, ^**^p < 0.01 and ^***^p < 0.001 (A: Friedman test, Dunn’s multiple comparison and B: Mann-Whitney).

We evaluated the expression of IL-20 subfamily cytokines in patients diagnosed for PPD-induced ACD. Before patch testing with the commercial allergen PPD, skin biopsies of uninvolved skin were collected (mentioned as PPD 0 hour). 8 hours, 24 hours and 48 hours after PPD application, allergic reactions were evaluated and skin biopsies were performed. 7 out of 11 patients presented a positive patch test after 24 hours, 3 patients only after 48 hours and for one patient tests remained negative even after 72 hours (Suppl. Table 1). As shown in Fig. 1, the expression of *IL19*, *IL20*, *Il22* and *IL24* was increased after PPD patch application and expression correlated with clinical observations. The increased expression of these cytokines in biopsies was less pronounced and clearly delayed in patients who showed no clinical signs at 24 hours (grey curves in Fig. 1A) compared with the 7 most rapidly affected patients (black curves in Fig. 1A). *IL24* was also induced, upon *in vitro* stimulation, in PBMCs of allergic patients compared to PBMCs of healthy controls. In contrast, the expression of *IL19* and *IL22* was similar in PBMCs of healthy controls compared to PBMCs of allergic patients, where *IL20* was not detectable (Fig. 1B). Altogether these data suggest that the IL-20-related cytokines might play a role in PPD-induced ACD. To examine the role of these cytokines in ACD, we developed a mouse model adapted from two other models^26,27^.

### Development of a mouse model of PPD-induced ACD

Mice treated with PPD showed a significant ear thickening starting after the third PPD application (Suppl. Fig. 1A). As observed in Suppl. Fig. 1A, mice that did not undergo the two first applications did not develop any ear thickening, indicating that preliminary sensitization is essential for PPD-induced CHS development. In addition, *Rag2*^−/−^ mice did not respond to PPD treatment in contrast to WT mice (Suppl. Fig. 1B). We also observed an increase in IgE level in sera from mice treated with PPD compared with vehicle control mice (VC), demonstrating the presence of an allergic reaction in our model (Suppl. Fig. 1C). At histological level, PPD-treated ear sections displayed the distinctive features of human ACD such as edema, neutrophilic infiltrate, dermal inflammatory infiltrate, exocytosis and acanthosis (Suppl. Fig. 1D). Moreover, in contrast to vehicle control mice, mice treated with PPD showed an infiltration of TCRβ^+^ cells in the dermis and epidermis, as in human ACD (Suppl. Fig. 1E).

### Upregulation of the expression of IL-20-related cytokines upon PPD treatment

**Figure 2:**
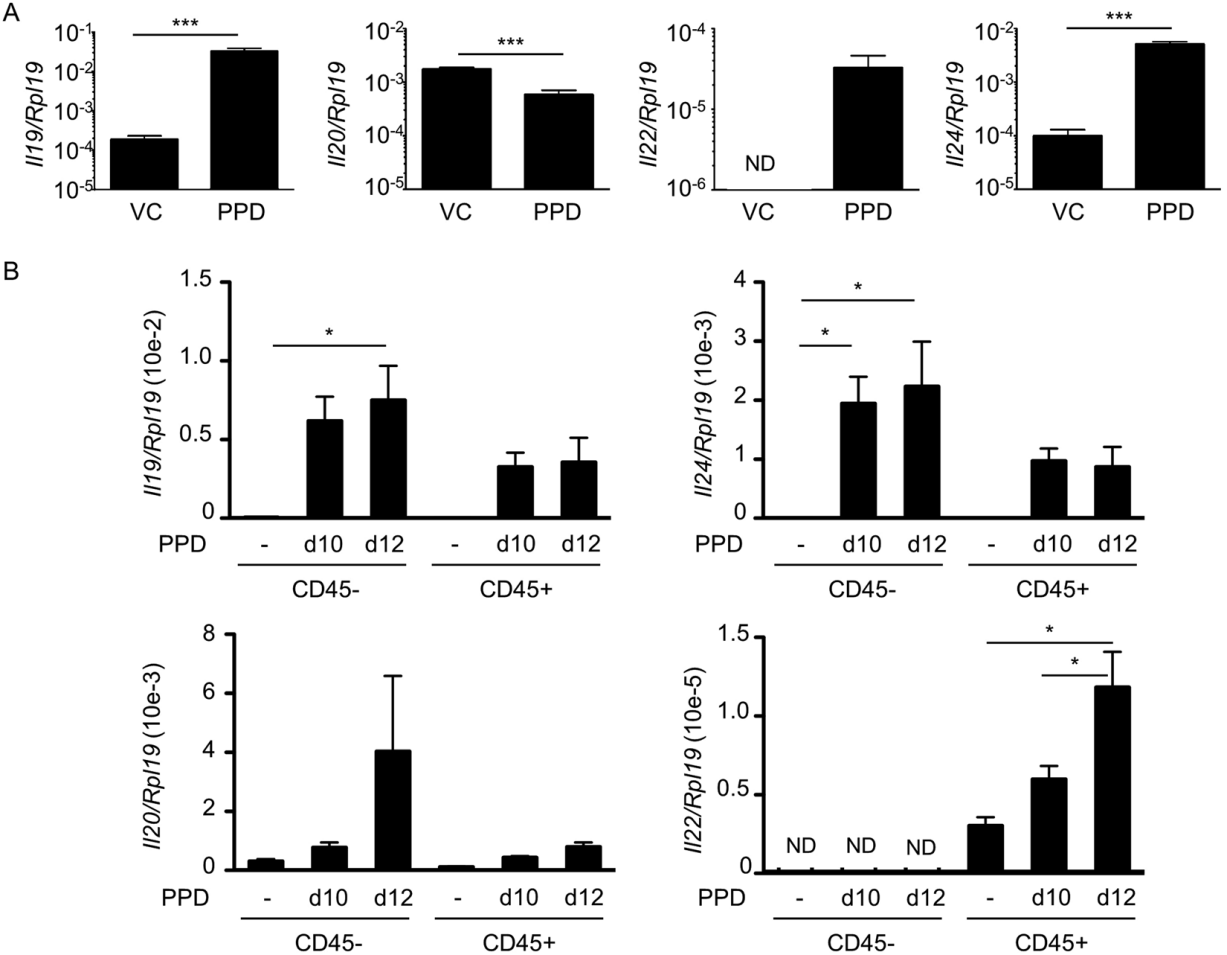
Expression of IL-20-related cytokines is increased in a mouse model of PPD-induced allergic contact dermatitis. 129/Sv mice were treated with PPD solutions. Quantitative RT-PCR analysis was performed for each indicated gene. (**A**) RNA was isolated from the total ear 24 hours after the second application. (**B**) 24 hours after the third (day 10) and the fifth (day 12) application, CD45^+^ cells were purified from the epidermis by MACS. RNA was isolated from both the CD45-positive and CD45-negative fraction. Data correspond to the mean ± SEM (N = 4 mice per group). Data are representative of three independent experiments. ^*^p < 0.05 and ^***^p < 0.001 (A: Mann-Whitney and B: one-way ANOVA, Bonferroni multiple comparison). ND: not detected, VC: vehicle control.

First, expression of IL-20-related cytokines was examined in this model on the total skin. During the early phase (24 hours after the second application), we observed an induction of *Il19*, *Il22* and *Il24* mRNA expression after PPD application compared with control skin, whereas *Il20* expression was decreased by PPD treatment (Fig. 2A). Expression of *Il19* and *Il24* was also induced during the late phase (24 hours after the third application) in contrast to *Il20* expression or *Il22* expression, which is not detected (Suppl. Fig. 2A). To determine whether hematopoietic cells or keratinocytes represent the main source of these cytokines, we purified CD45^+^ and CD45^−^ cells from the epidermis (Suppl. Fig. 2B). As expected, *Cd3e* expression was restricted to the CD45-positive fraction and increased after PPD challenges, reflecting the T cell infiltration observed by flow cytometry (Suppl. Fig. 2C). *Krt10*, a keratinocyte marker, was mainly expressed in the CD45-negative fraction, confirming enrichment of keratinocytes in this fraction (Suppl. Fig. 2C). Interestingly, *Il19* and *Il24* expression appeared to be upregulated by PPD treatment in both fractions, although statistical significance was reached only in the CD45-negative fraction (Fig. 2B). *Il20* was not significantly affected and *Il22* expression was only upregulated in CD45-positive cells, at a later time point (at day 12, after five PPD applications) (Fig. 2B).

Taken together these results indicate that expression of *Il19*, *Il22* and *Il24* are quickly upregulated after PPD treatment, and non-hematopoietic cells represent the main source of IL-19 and IL-24 whereas CD45^+^ cells produce IL-22.

### Il22ra1-, Il20rb and Il24-deficient mice are partially protected against PPD-induced CHS

**Figure 3:**
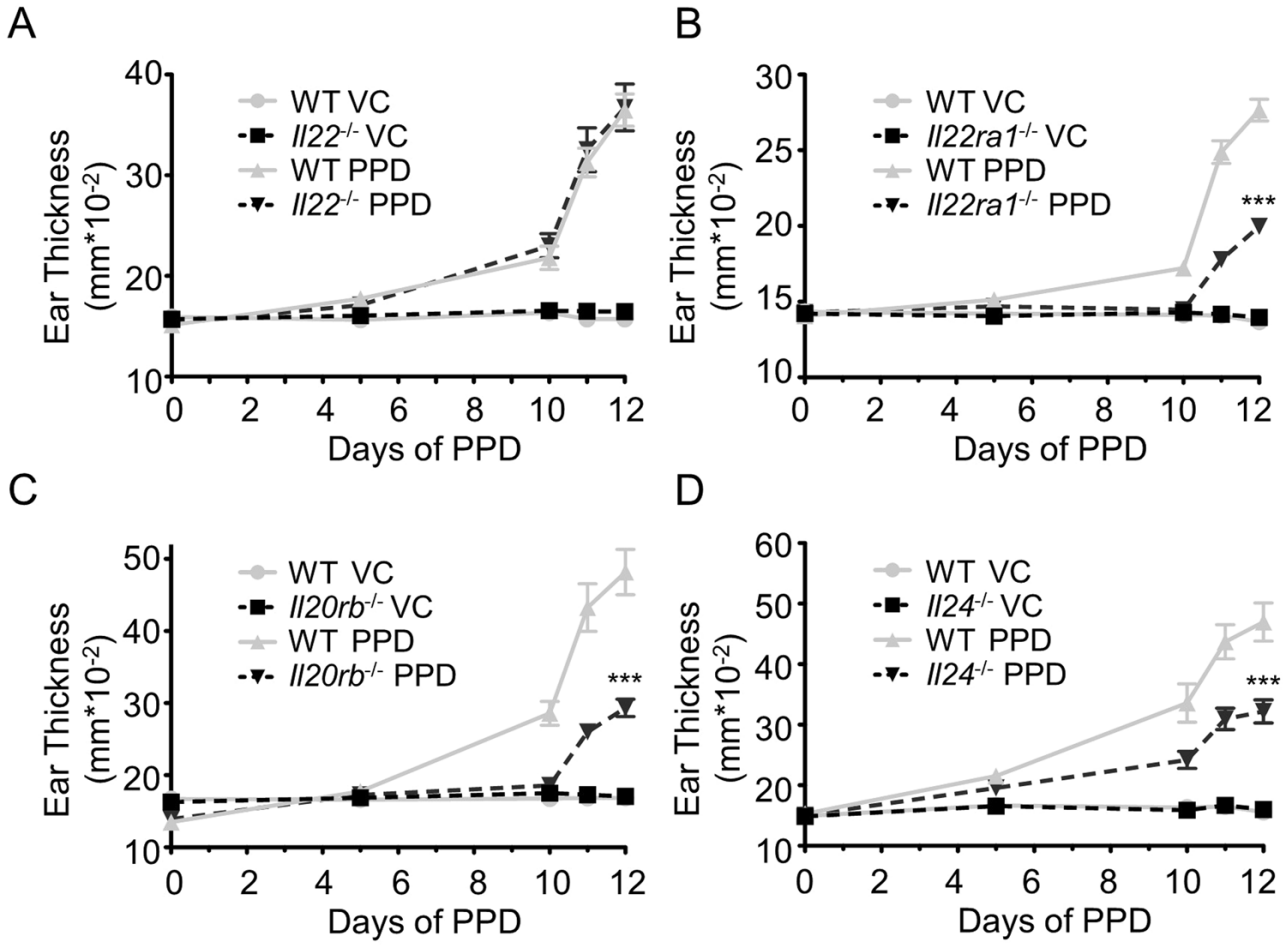
*Il24*^−/−^, *Il22ra1*^−/−^ and *Il20rb*^−/−^ mice, but not *Il22*^−/−^ mice, are partially protected against CHS induced by PPD. Ear thickness was measured before each PPD treatment and 24 hours after the last application with a micrometer screw to evaluate the development of CHS. (**A**) Ear thickness in *Il22*^+/+^ and *Il22*^−/−^ 129/Sv mice. (**B**) Ear thickness in *Il22ra1*^+/+^ and *Il22ra1*^−/−^ 129/Sv mice. (**C**) Ear thickness in *Il20rb*^+/+^ and *Il20rb*^−/−^ C57BL/6 mice. (**D**) Ear thickness in *Il24*^+/+^ and *Il24*^−/−^ C57BL/6 mice. Data are means ± SEM (N = at least 4 for VC groups and N = at least 5 for PPD-treated groups) and representative of at least three independent experiments ^***^p < 0.001 Comparison of PPD treated mice (two-way Anova, Bonferroni multiple comparison). VC: vehicle control.

**Figure 4:**
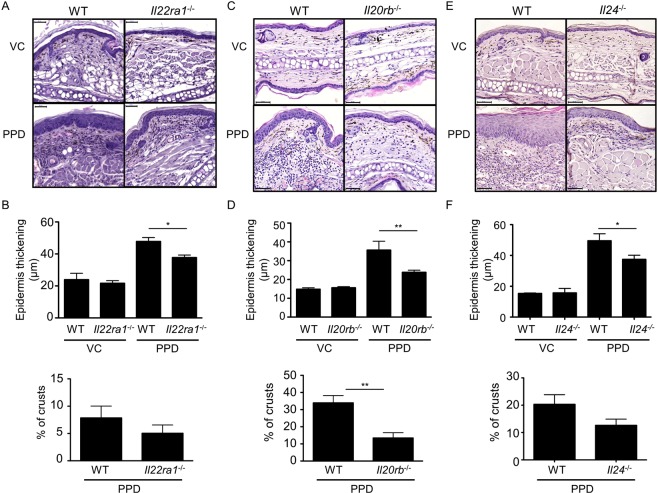
Acanthosis is less prominent in *Il24*^−/−^, *Il22ra1*^−/−^ and *Il20rb*^−/−^ mice compared with WT mice after PPD treatment. (**A**,**C**,**E**) HE staining of ear skin sections from mice, treated or not with PPD, 24 hours after the sixth application. One representative picture is shown for each treatment regimen (original magnification x30, scale bar = 50 μm). (**B**,**D**,**F**) Acanthosis was evaluated by measuring the epidermal thickness at six different places by using Pannoramic viewer measuring tool. The percentages of crusts are calculated by dividing the length of crusts by the length of the section. These analyses were performed in *Il22ra1*^+/+^ and *Il22ra1*^−/−^ 129/Sv mice (**A**,**B**), in *Il20rb*^+/+^ and *Il20rb*^−/−^ C57BL/6 mice (**C**,**D**) and *Il24*^+/+^ and *Il24*^−/−^ C57BL/6 mice (**E**,**F**). Data are means ± SEM (N = 5 for VC groups and N = 8 for PPD-treated groups) and representative of four independent experiments. ^*^p < 0.05 and ^**^p < 0.01 (Mann-Whitney to compare treated mice). Histological analysis was performed by two evaluators. VC: vehicle control.

To analyze the role of these IL-20 subfamily cytokines in CHS, we treated *Il22-*, *Il24-*, *Il22ra1-* and *Il20rb*-deficient mice with PPD. *Il22*-deficient mice showed a similar ear thickening as WT mice (Fig. 3A). In contrast, *Il22ra1*-deficient mice showed a partial protection against the development of CHS displaying less ear swelling compared with PPD-treated WT littermates (Fig. 3B), as well as less acanthosis (Fig. 4A,B). This observation suggested that IL-22R plays a role in this model, independently of IL-22 activity. As *Il24* expression is strongly increased upon PPD treatment and IL-22R can associate with IL-20R2 to form a receptor complex for IL-24, we hypothesized that *Il24*- and *Il20rb*-deficient mice might be protected in the same way as *Il22ra1*-deficient mice. As shown in Fig. 3C and D, *Il24*- and *Il20rb*-deficient mice showed a partial protection, which is associated with less acanthosis (Fig. 4C-F) and a smaller proportion of crusts (Fig. 4C-F) compared to WT littermate mice.

### The absence of IL-24 activity does not affect the IgE-dependent allergy but partially prevents the epidermal infiltration of CD45^+^ cells and neutrophils induced by PPD

**Figure 5:**
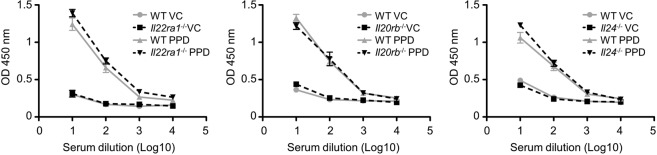
IgE-dependent allergy is similar in WT and *Il22ra1*^−/−^, *Il20rb*^−/−^ and *Il24*^−/−^ mice after PPD treatment. Mice were treated or not with PPD and 24 hours after the third application, IgE levels in the sera were assessed by ELISA. This analysis was performed in *Il22ra1*^+/+^ vs *Il22ra1*^−/−^ 129/Sv mice (left panel), in *Il20rb*^+/+^ vs *Il20rb*^−/−^ C57BL/6 mice (central panel) and in *Il24*^+/+^ and *Il24*^−/−^ C57BL/6 mice (right panel). Data are means ± SEM (N = 3 for VC groups and N = 6 for PPD-treated groups). VC: vehicle control.

**Figure 6:**
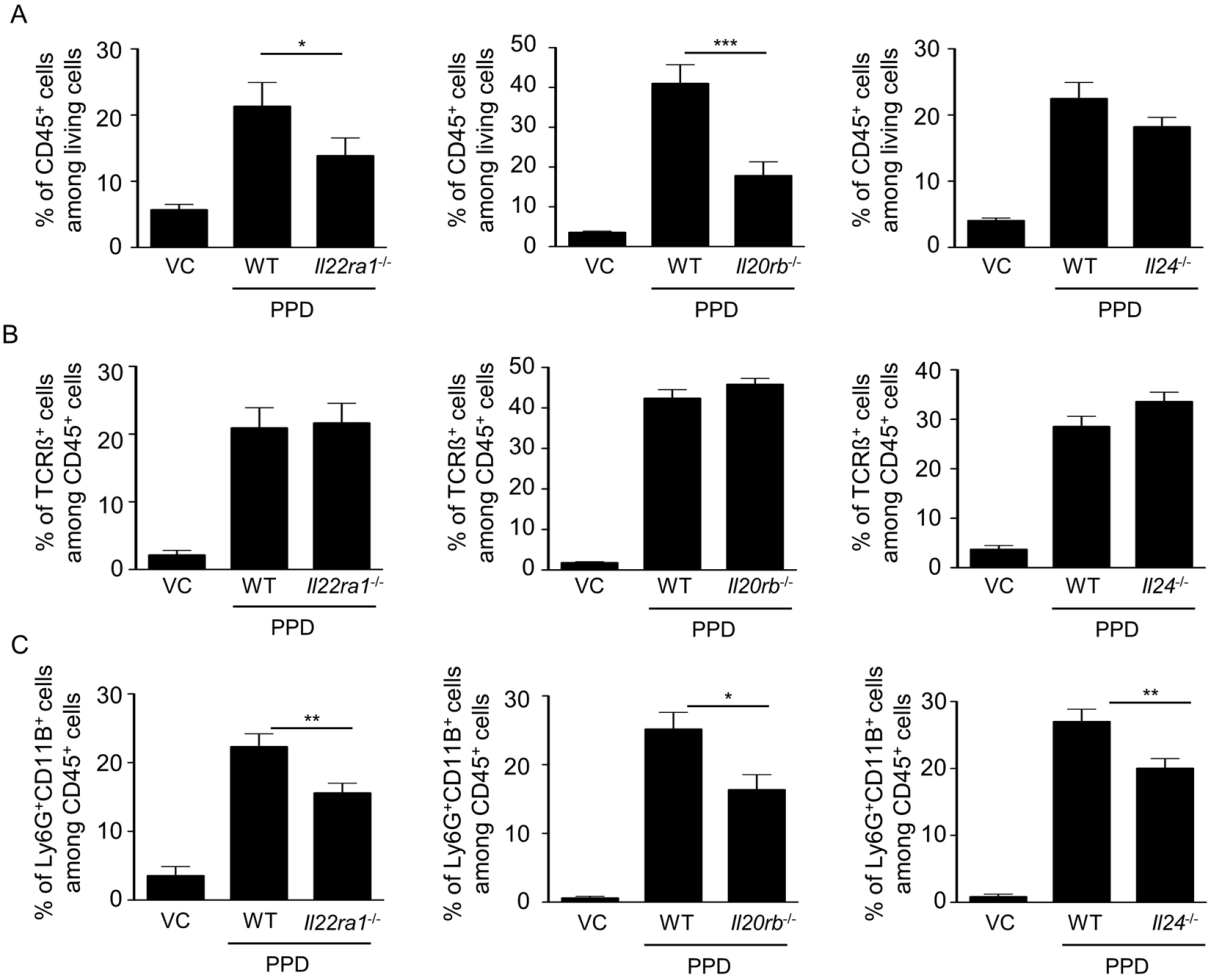
Epidermal infiltration of CD45^+^ cells decreases in *Il22ra1*^−/−^ and *Il20rb*^−/−^ mice compared with WT mice. Flow cytometry on epidermal cells from mice, treated or not with PPD (VC), 24 hours after the second application. (**A**) The percentage of CD45^+^ cells among living cells was analyzed. (**B**) The proportion of TCRß^+^ cells among CD45^+^ living cells was analyzed. (**C**) The proportion of Ly6G^+^CD11B^+^ cells among CD45^+^ living cells was analyzed. These analyses were performed in *Il22ra1*^+/+^ and *Il22ra1*^−/−^ 129/Sv mice (left panels), in *Il20rb*^+/+^ and *Il20rb*^−/−^ C57BL/6 mice (central panels) and *Il24*^+/+^ and *Il24*^−/−^ C57BL/6 mice (right panels). Data are means ± SEM (N = 5 for VC groups and PPD-treated groups) and representative of three independent experiments. ^*^p < 0.05 and ^***^p < 0.001 (Mann-Whitney to compare treated mice). VC: vehicle control.

**Figure 7:**
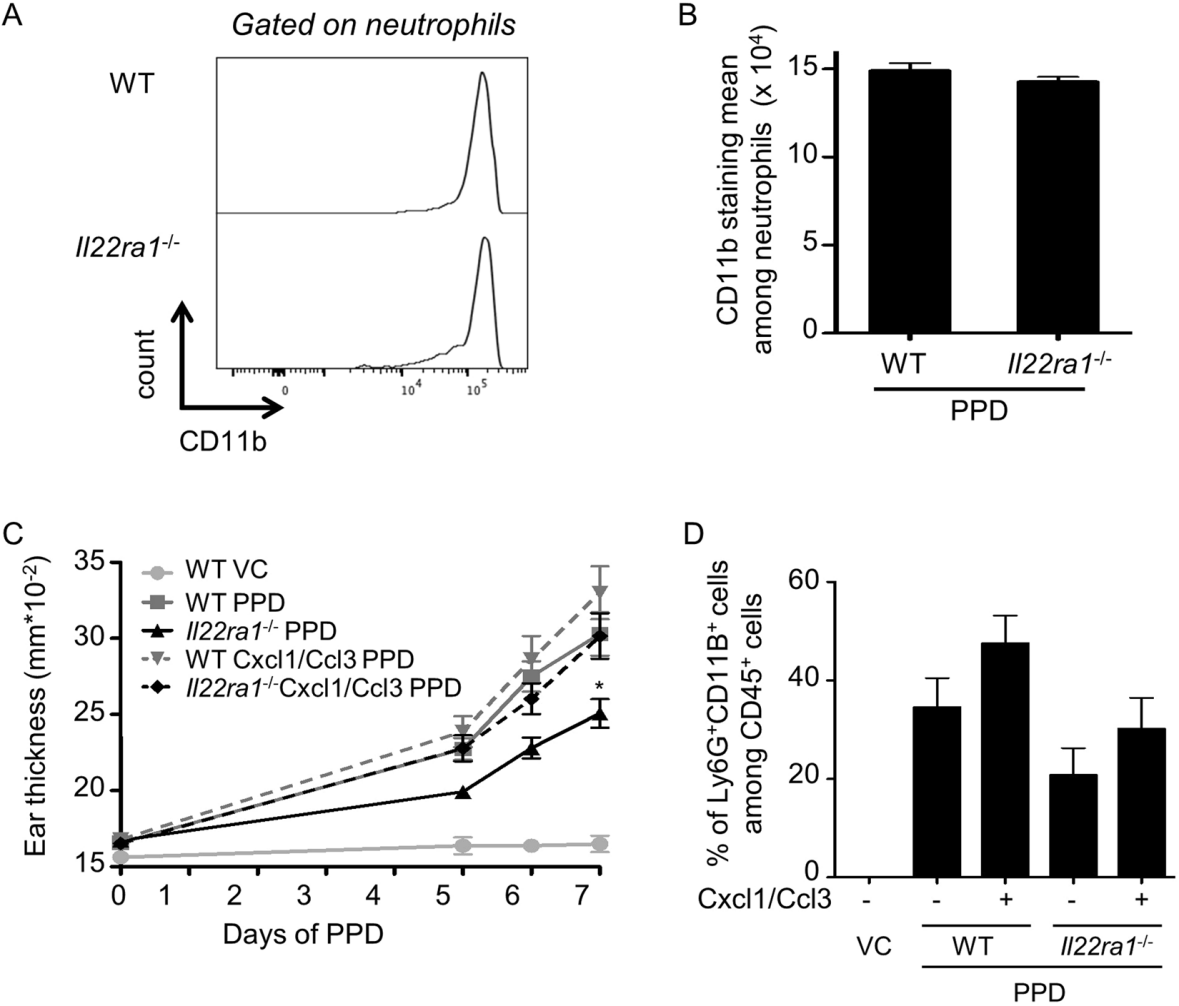
Neutrophil activity is similar in WT and *Il22ra1*^−/−^ mice but induction of neutrophil recruitment increases ear thickness. (**A**,**B**) After two PPD applications, ears from *Il22ra1*^+/+^ and *Il22ra1*^−/−^ C57BL/6 mice were collected to analyze neutrophil population by flow cytometry. (**A**) CD11B staining in neutrophil population (gated on Ly6G^+^ CD11B^+^ cells among CD45^+^ living cells) from *Il22ra1*^+/+^ and *Il22ra1*^−/−^ mice after PPD treatment (one representative mouse per group). (**B**) Mean of CD11B staining among neutrophil populations. Data are means ± SEM (N = at least 5 mice per group) and representative of two independent experiments. (**C**,**D**) Cxcl1 and Ccl3 chemokines were injected in ears during first and second applications to induce neutrophil recruitment and flow cytometry analysis was performed on epidermal cells from mice, treated or not with PPD (VC), 48 hours after the second application. PBS was injected in mice without chemokine injection. This analysis was performed in *Il22ra1*^+/+^ vs *Il22ra1*^−/−^ C57BL/6 mice. (**C**) Ear thickness was measured before each PPD application and each day after the second application. (**B**) The proportion of Ly6G^+^CD11B^+^ cells among CD45^+^ living cells was analyzed. Data are means ± SEM (N = 4 for VC group and at least 5 for PPD-treated groups) and representative of three independent experiments. ^*^p < 0.05 (C: two-way Anova, Bonferroni multiple comparison). VC: vehicle control.

To analyze the allergic process, we measured the IgE levels in the sera of *Il20rb-*, *Il24-*, *Il22ra1*-deficient mice. We showed similar IgE level than WT mice (Fig. 5), suggesting that the IgE-dependent allergy was still present in deficient mice. We then examined the immune cells infiltrate in the ear skin and we observed that the proportion of CD45^+^ cells was increased after PPD treatment in the epidermis of WT mice and to a lesser extent in *Il20rb-*, *Il24-*, *Il22ra1-*deficient mice (Fig. 6A). We observed similar results in the dermis (Suppl. Fig. 3A). As TCRß^+^ cells are known to play a role in CHS, we studied the proportion of TCRß^+^ cells after PPD application. The percentage of TCRß^+^ cells was increased in the dermis and epidermis of PPD-treated mice but no difference was observed between WT and *Il20rb-*, *Il24-*, *Il22ra1-*deficient mice (Fig. 6B and Suppl. Fig. 3B). Another population present at a high percentage (20–30%) after PPD treatment is Ly6G^+^CD11B^+^ cells, which are neutrophils (Fig. 6C). In both epidermis and dermis, the percentage of Ly6G^+^CD11B^+^ was significantly lower in *Il20rb-*, *Il24-*, *Il22ra1*-deficient mice compared to WT mice (Fig. 6C and Suppl. Fig. 3C). We hypothesized that this difference in neutrophil infiltrate could explain the protective effect observed in deficient mice. We did not observe any difference in neutrophil activity based on CD11B staining between neutrophils from WT versus *Il22ra1*-deficient mice (Fig. 7A,B). However, when we induced neutrophil recruitment in ears by injecting a cocktail of Cxcl1 and Ccl3, two chemokines known to be induced by IL-22R and able to induce neutrophil recruitment, we observed a significant increase in ear thickening (Fig. 7C). We confirmed that ear thickness was correlated with the percentage of neutrophils in the epidermis, because this percentage was higher after chemokine injection (Fig. 7D). In addition, we noticed that the significant decrease in the immune cell infiltrate in deficient mice happened during the early phase of PPD treatment but not during the late phase (Suppl. Fig. 4), suggesting that IL-24 is involved in the early phase of the inflammatory reaction. Moreover, in auricular lymph nodes, we did not observe any difference in cell number, cytokine expression (*Il4*, *Ifng* and *Il17*) and cell composition (B220, CD3, CD4 and CD8) in *Il20rb-*, *Il24-*,and *Il22ra1*-deficient mice compared to WT mice (Suppl. Figs 5 and 6). These data suggest that protection in the absence of IL-24 occurs mainly in the skin and is not associated with global inflammation. Together, these observations indicate that IL-24 plays a role in the local, early inflammatory reaction induced by PPD application. It induces acanthosis and increases the inflammatory infiltrate, particularly neutrophils, without affecting IgE-dependent allergy.

## Discussion

Our results indicate that the IL-20-related cytokines are markers of PPD allergy because we found a correlation between IL-20-related cytokine expression and the severity of reactions in patients. We also demonstrated that IL-20-related cytokines, and more particularly IL-24, play a role in PPD-induced CHS since *Il24*-, *Il22ra1*- and *Il20rb* deficient mice were partially protected against the development of acanthosis and the neutrophil influx in our mouse model.

Here, we have developed a mouse model that recapitulates the typical features of PPD-induced ACD with respect to spongiosis, exocytosis and inflammatory infiltrate. As expected in murine CHS model, the sensitization phase takes 5–7 days whereas in human it takes 10–15 days^28^. After 6 days, we already observed a massive infiltrate of αβ T cells in PPD-treated skin in contrast to other skin diseases, such as the imiquimod-induced psoriasis model where γδ T cells play an important role^16,29^. Our data indicate that the PPD-induced CHS model is associated with a Th2 response because mice treated with PPD have an increased expression of *Il4* in the skin (data not shown) and a high IgE blood level. In addition, we observed an important induction of *Il19* and *Il24* expression, which are both Th2-associated cytokines^30,31^. Different studies have already shown that repeated elicitation is associated with a Th2 response, while acute ACD is rather Th1-dominated^32,33^. In contrast, we observed no *Il17* expression (data not shown) after PPD application suggesting that Th17 cells do not play a major role in our CHS model, while IL-17 plays an important role in DNFB and TNCB-induced CHS^34,35^. High levels of Th17 cytokines were also detected in the blood and in the skin of patients suffering from nickel-induced contact dermatitis demonstrating the association of Th17 and ACD with some allergens^20,21,36^.

*Il24*-, *Il22ra1*- and *Il20rb*-deficient mice are partially protected against PPD-induced CHS, demonstrating that IL-20-related cytokines and more particularly IL-24 play a detrimental role in CHS. Along the same line, IL-20R1 was described to play a role in DNFB-induced CHS because ear swelling was decreased in mice receiving an anti-IL-20R1 blocking antibody^37^ suggesting that IL-20-related cytokines aggravate DNFB-induced CHS. These and our results are not consistent with a previous study that showed a more prominent CHS reaction in *Il20rb*-deficient mice than in WT mice in an acute model of TNCB-induced CHS, suggesting that IL-19, IL-20 and/or IL-24 play a beneficial role in that model^25^. This discrepancy could be due to the fact that the kinetics used by them is different from ours and consists of one sensitization followed by a unique challenge 5 days later. Alternatively, IL-20-related cytokines might play a beneficial role in Th17-associated TNCB models.

Our results demonstrate that IL-24, but not IL-22, is required for the skin reaction induced by PPD application. However, we cannot exclude that IL-19 and IL-20, which act via IL-20R2 and are highly expressed after PPD application in allergic patients, could also play a role in the development of PPD-induced CHS. Nevertheless, *Il20rb*-deficient mice showed the same level of protection as *Il22ra1*^−/−^ mice, suggesting that IL-19 does not play a major role in this model. In line with this result, a study demonstrated that IL-19 blood level correlates with disease severity in psoriasis but IL-19 alone has only few effects on keratinocyte proliferation and migration. Instead, IL-19 strengthens the action of IL-17A by amplifying the expression of antimicrobial peptides and neutrophil-attracting chemokines^37^. As IL-17 is not produced in our model of PPD-induced CHS, the role of IL-19 is unlikely. In contrast, based on the more prominent protection in *Il20rb*-deficient mice compared to *Il24*-deficient mice, IL-20, which is constitutively expressed in our model, could indeed play a role. Of note, IL-20 has proinflammatory roles in autoimmune diseases such as, psoriasis and rheumatoid arthritis. IL-20 induces epidermal hyperplasia and inhibits terminal keratinocyte differentiation in human epidermis^38,39^. IL-20 also induces the production of proinflammatory cytokines, including TNF-α and IL-1β, by synovial fibroblasts^40,41^.

Neutrophil influxes were lower in the epidermis and dermis of *Il24*-, *Il22ra1*- and *Il20rb* deficient mice compared to WT littermates, in line with lower levels of chemokine associated with neutrophil recruitment such as Cxcl3, Ccl3 or Cxcl5 (data not shown). We confirmed the role of neutrophils in ear thickening in our model by injecting Cxcl1 and Ccl3 chemokines in mice ears. The role of neutrophils was also highlighted in a study that reported the importance of neutrophils during both sensitization and elicitation phases of CHS^42^. Neutrophils are required for the CHS response because absence of neutrophils during sensitization phase abrogates ear thickness and inflammatory response^42^.

In conclusion, in contrast to psoriasis where different IL-20 related cytokines play a role, IL-24 is the main IL-20-related cytokine playing a role in PPD-induced CHS, most probably via its effect on keratinocytes. It induces acanthosis and production of chemokines, in turn triggering a neutrophil influx that plays a crucial role in contact hypersensitivity.

## Material and Methods

### Patients

Eleven patients with a history of positive patch-test reaction to PPD were included in this study. The patients were otherwise healthy and only investigated when clinically in remission of their dermatitis. All of them had a history of allergic contact dermatitis after using hair dyes or after temporary black henna tattoos. The study and data accumulation were conducted with the approval of the Institutional Ethical Committee, Commission d’Ethique Biomédicale Hospitalo-Facultaire de l’Université catholique de Louvain (NCT 340320084407). All experiments were performed in accordance with relevant guidelines and regulations. Informed consent for all the diagnostic procedures was obtained from all study subjects.

All subjects were examined clinically and patch tested with para-phenylenediamine 1% diluted in petrolatum (Chemotechnique). Three series of PPD patch tests were applied. The patch-test materials used were IQ Ultra^®^ chambers (Chemotechnique) covered on the buttockx with Fixomull stretch^®^ (Smith and Nephew). The patch-test reactions were evaluated after 8, 24 and 48 hours according to the ICDRG criteria (Suppl. Table 1)^43^. Three mm-punch biopsies from patch tests, whether positive or negative, were collected at 8, 24, and 48 hours following PPD application. Before patch testing (0 hour), normal skin was also biopsied.

### PBMCs isolation and stimulation

Blood samples were collected before patch testing. Total human PBMCs were purified from the blood of control or allergic patients by centrifugation on a Lymphoprep gradient (Elitech). Cells were then washed with PBS EDTA 1 mM and resuspended in autologous medium (RPMI medium (Gibco) containing 5% of plasma patient). PBMCs were stimulated during 48 hours at 37 °C with anti-CD3 anti-CD28 beads (Life, 500 000 beads for 10^6^ cells) and PPD (Sigma, 2.5 µg/mL). After this incubation, cells were harvested for RNA extraction.

### Mice

All mice used in this study were bred in the animal facility of the Brussels branch of the Ludwig Institute for Cancer Research under specific pathogen-free conditions. *Rag2*^−/−^ BALB/c mice were originally purchased from Taconic and C57BL/6NJ *Il24*^−/−^ mice were purchased from Jackson laboratory. *Il20rb*^−/−^ mice, in C57BL/6 background, were provided by U.M. Wegenka (University Medical Center, Ulm, Germany)^25^. Wild-type (WT) 129/Sv mice were originally purchased from Harlan. *Il22*–deficient mice were generated in 129/Sv background in our laboratory as described previously^44^. IL-22R-deficient mice were generated in 129/Sv and C57BL/6 background in our laboratory as described in Suppl. Fig. 7. All mice were bred as heterozygous and littermate controls were used for *in vivo* experiments. The *Il22*^−/−^ mice were used in 129/Sv background, *Il22ra1*^−/−^ mice were used in 129/Sv or C57BL/6 background and *Il24*^−/−^ and *Il20rb*^−/−^ in C57BL/6 background. The experiments were performed in compliance with institutional guidelines and were approved by the Animal Research Ethical Committee of the Université catholique de Louvain (2015/UCL/MD/09). Mice between 8 and 12 weeks of age were shaved on the back skin one day before CHS triggering.

### CHS model

Our CHS model is based on the timing used in Rothe *et al*. study^26^, namely PPD application at day 0, 5, 10, 11, 12 and 13. For the first and the second application, mice were treated on shaved back skin and the dorsum side of ears by applying a solution of H_2_O_2_ 3% and PPD (CAS 106–50–03, Sigma), 3% [W/V] for 129/Sv background mice and 5% for C57BL/6 and BALB/c background mice, diluted in acetone: olive oil (4:1). The application of PPD on ears from the first application aims to mimic what happens in PPD-allergic patients who are both sensitized and elicitated at the same site. The third application is done by applying the H_2_O_2_/PPD solution on ears only. The next applications are performed without H_2_O_2_. Control mice received vehicle solution (including H_2_O_2_ 3% for the three first applications). All solutions were prepared freshly. Ear thickness was measured before each application of PPD and 24 hours after the last application with a micrometer screw (Mitutoyo). For chemokine injection, we injected 1 µg Cxcl1 and 3 µg Ccl3 (Immunotools) in ears during the first and the second applications.

### Single-cell suspension and FACS staining

Ears were dissected and incubated overnight in dispase II at 1 U/ml (Roche) at 4 °C. The epidermis and the dermis were separated as previously described^45^. Cells were incubated with 10 μg/ml of purified rat anti-mouse CD16-CD32 monoclonal antibody (Fc Block). Then, the specific antibodies were added for 1 h at 2 μg/ml at 4 °C: PercP-labeled anti-CD45 (30-F11), APC-labeled anti-TCRβ (H57–597), PE-labeled anti-CD11b (M1/70), FITC-labeled anti-Ly6G (1A8). A viability marker was also added (LIVE/DEAD® Fixable Near-IR Dead Cell Stain Kit, Life). Cells were gated, based on forward and side scatter, on viability marker and on living hematopoietic cells (CD45^+^ cells) with FACS Fortessa (BD Biosciences). Postacquisition analysis was performed using FlowJo software (Tree Star).

### Purification of CD45^+^ cells

Epidermal cell suspensions from 129/Sv mice were prepared as described above. The MACS system from Miltenyi Biotec was used to isolate CD45^+^ cells, following the manufacturer’s instructions. Briefly, cells were incubated for 20 min at 4 °C with anti-CD45 antibody-coupled microbeads, washed, and separated by two passages on the MACS instrument. Purification was checked by FACS analysis with an anti-CD45 antibody and determined to be at least 90% of purity for PPD-treated skin.

### IgE measurement

IgE titers were measured in sera by ELISA using specific reagents from LO/IMEX, (Université catholique de Louvain). All absorbance reads were made at 450 nm, using a 96-well plate spectrophotometer.

### Histological analysis

Paraffin tissue blocks of mouse ear skin were prepared using routine methods and consecutive sections were made. The sections were stained with HE for mouse skin and scanned with Mirax (Zeiss). Epidermal thickness was measured at different places of the section thanks to Pannoramic Viewer measuring tool (3DHISTECH). Percentages of crusts were calculated by measuring length of crusts divided by the length of the section. Two evaluators performed analysis of the staining.

### RT-PCR

Total RNA was isolated from mouse ears or skin of patients using TriPure isolation reagent (Roche). Reverse transcription and condition used for the RT-qPCR were described before^45^. Quantitative PCR (qPCR) amplifications were performed using primer sets and TaqMan probes corresponding to murine β-*actin*, *Il19*, *Il22*, *Il24, Ifng, Il4, Il17* and Ngp or human *EF1, IL19, IL20, IL24* with qPCR Mastemix TaqMan (Eurogentec). For murine *Rlp19*, *Il20*, *Cd3e* and *Krt10*, qPCR was done using MasterMix for SYBR Green (Eurogentec). The sequences of primers and probes are listed in Suppl. Table 2.

### Statistics

Results are presented as the mean ± SEM. Statistical significance between groups was assessed by using one-tailed unpaired Student t test, Mann-Whitney test in non-parametric conditions and two-way ANOVA with Bonferroni’s post-test for the ear thickening curves, using the Prism software (GraphPad software).

## Supplementary information

Suppl. Information
