# Supplementary material for: IL-24 contributes to skin inflammation in Para-Phenylenediamine-induced contact hypersensitivity
Source: Sci Rep. 2019 Feb 12;9:1852. doi: 10.1038/s41598-018-38156-4 (PMC6372603; doi:10.1038/s41598-018-38156-4)
Supplement: Supplementary file 1 — Suppl. Information [file 41598_2018_38156_MOESM1_ESM.pdf]

IL-24 contributes to skin inflammation in Para-Phenylenediamine-induced contact hypersensitivity

Astrid B. Van Belle, Perrine M. Cochez, Magali de Heusch, Lisa Pointner, Remi Opsomer, Peggy Raynaud, Younes Achouri, Emilie Hendrickx, Pamela Cheou, Guy Warnier, Jean-Christophe Renauld, Marie Baeck and Laure Dumoutier.

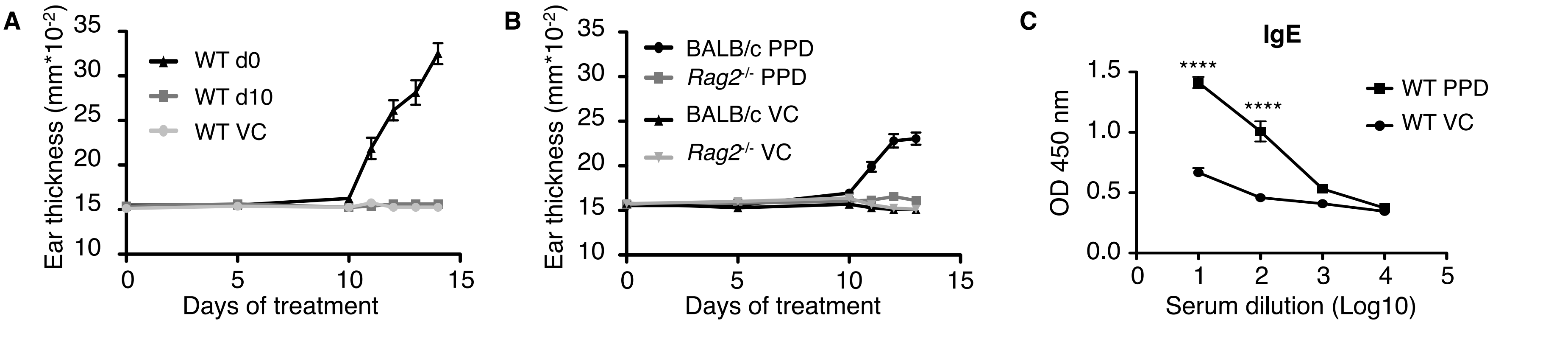

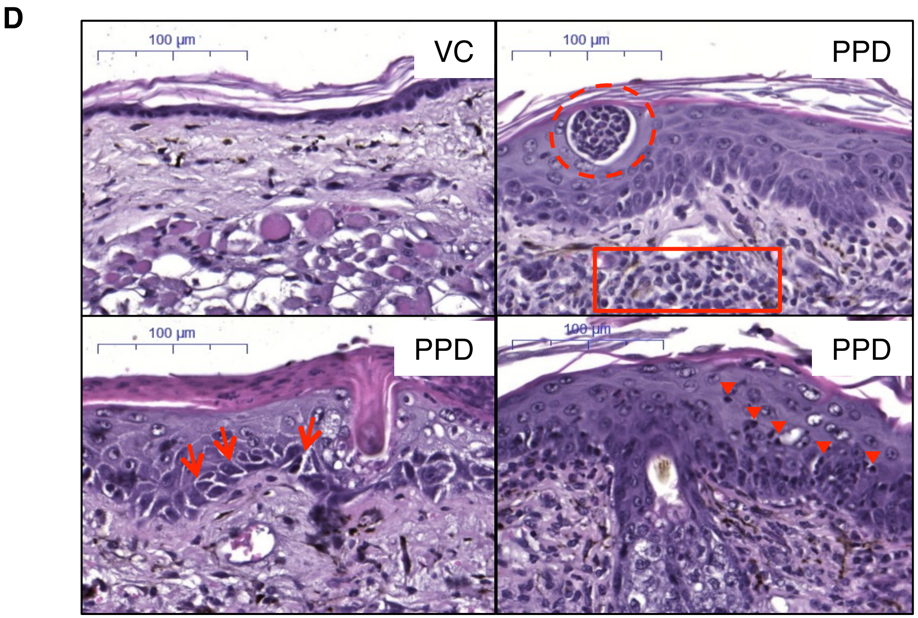

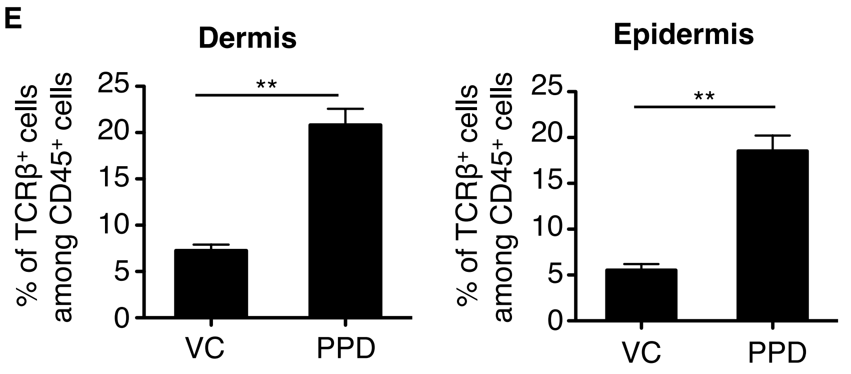

Suppl Figure 1: **Development of a mouse model of CHS.** A, Ear thickness was measured in C57BL/6 mice before each PPD treatment and 24 hours after the last PPD application with a micrometer screw to evaluate the development of CHS in mice that were treated with PPD solution from day 0 vs from day 10. Data are means ± SEM (N = 4 mice per group) and representative of four independent experiments. B, Ear thickness in BALB/c or *Rag*$2^{-/-}$ mice treated with PPD solution. Data are means ± SEM (N = at least 4 for VC groups and N = at least 9 for PPD-treated groups) and representative of one experiment. C, Immunoglobulin E levels in the sera of VC versus PPD-treated C57BL/6 mice were assessed by ELISA 24 hours after the third PPD application. Data are means ± SEM (N = 4 for WT VC and N = 8 for WT PPD). ****$p < 0.0001$ compared with PPD-treated WT mice (two-way Anova, Bonferroni multiple comparison). D, HE staining of VC and PPD-treated skin 24 hours after the sixth PPD application in C57BL/6 mice. Spongiotic areas (arrows), neutrophil infiltrate (dotted ellipse), inflammatory infiltrate (rectangle) and exocytosis (heads of arrow) are observed in PPD-treated WT mice. E, Flow cytometry on epidermal and dermal cells from VC and PPD-treated C57BL/6 skin 24 hours after the third PPD application. Cells were gated on $CD45^+$ living cells and expression of TCRβ was analyzed. Data are means ± SEM (N = at least 7 mice per group) and representative of four independent experiments. **$p < 0.01$ (Mann-Whytney). (VC = vehicle control).

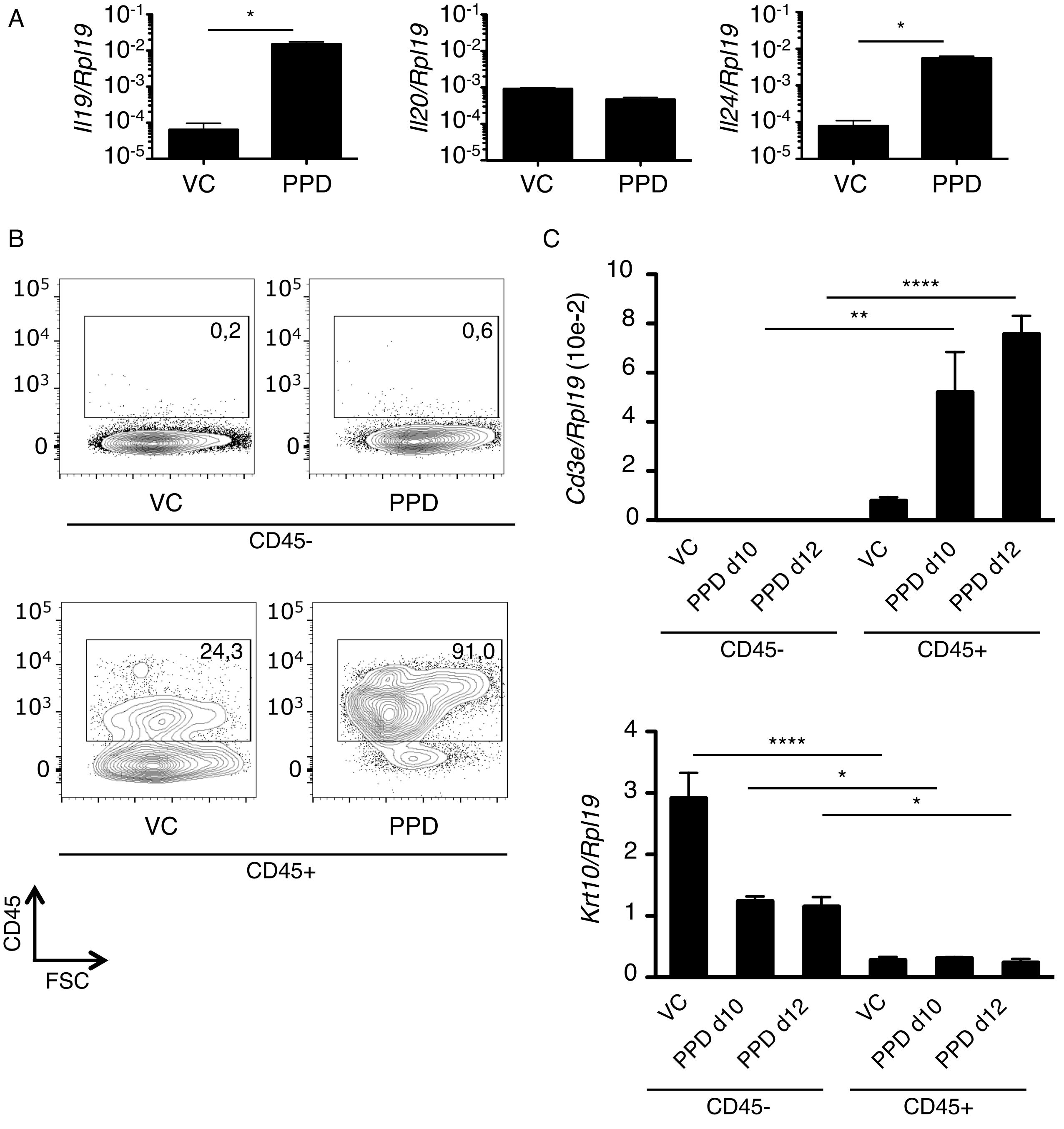

Suppl Figure 2: Cytokine expression after the third application and source of cytokines. 129/Sv mice were sensitized and challenged with PPD solutions. A, RNA was isolated from total ear 24 hours after the third PPD application and quantitative RT-PCR analysis was performed for *Il19*, *Il20* and *Il24*. B, $CD45^+$ cells were purified from the epidermis by MACS 24 hours after the third (day 10) and the fifth (day 12) PPD applications and CD45 expression was checked by flow cytometry in $CD45^-$ and $CD45^+$ fractions. C, RNA was isolated from both CD45-positive and CD45-negative fraction. Quantitative RT-PCR analysis was performed for *Cd3e* and *Krt10* genes. Data correspond to the mean ± SEM (N = 4 mice per group). Data are representative of three independent experiments. $^*p < 0.05$, $^{**}p < 0.01$ and $^{****}p < 0.0001$ (one-way Anova, Bonferroni multiple comparison). (VC = vehicle control).

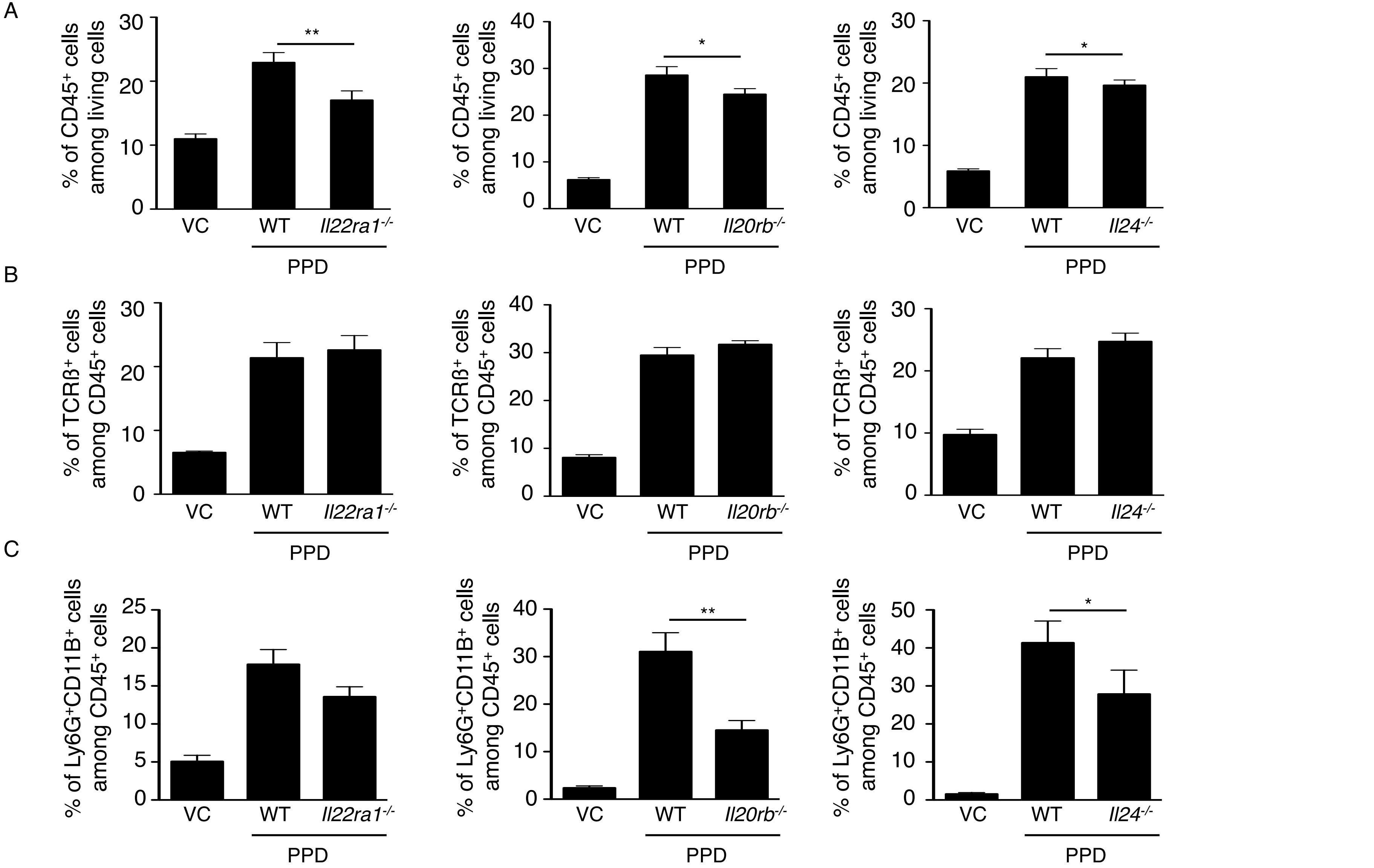

Suppl Figure 3: Inflammatory infiltrate is less prominent in the dermis of *Il22ra1*$^{-/-,}$ *Il20rb*$^{-/-}$ and *Il24*$^{-/-}$ mice during early phase. Flow cytometry on dermal cells from VC and PPD-treated mice. The skin was harvested 24 hours after the second PPD application. For CD45 (A), cells were gated on living cells and for TCRß (B) and Ly6G CD11B (C) cells were gated on CD45^{+} living cells. Data are means ± SEM (N = at least 5 mice per group) and representative of at least 2 independent experiments. *$p < 0.05$ and **$p < 0.01$ (Mann-Whitney to compare the two groups of treated mice). (VC = vehicle control).

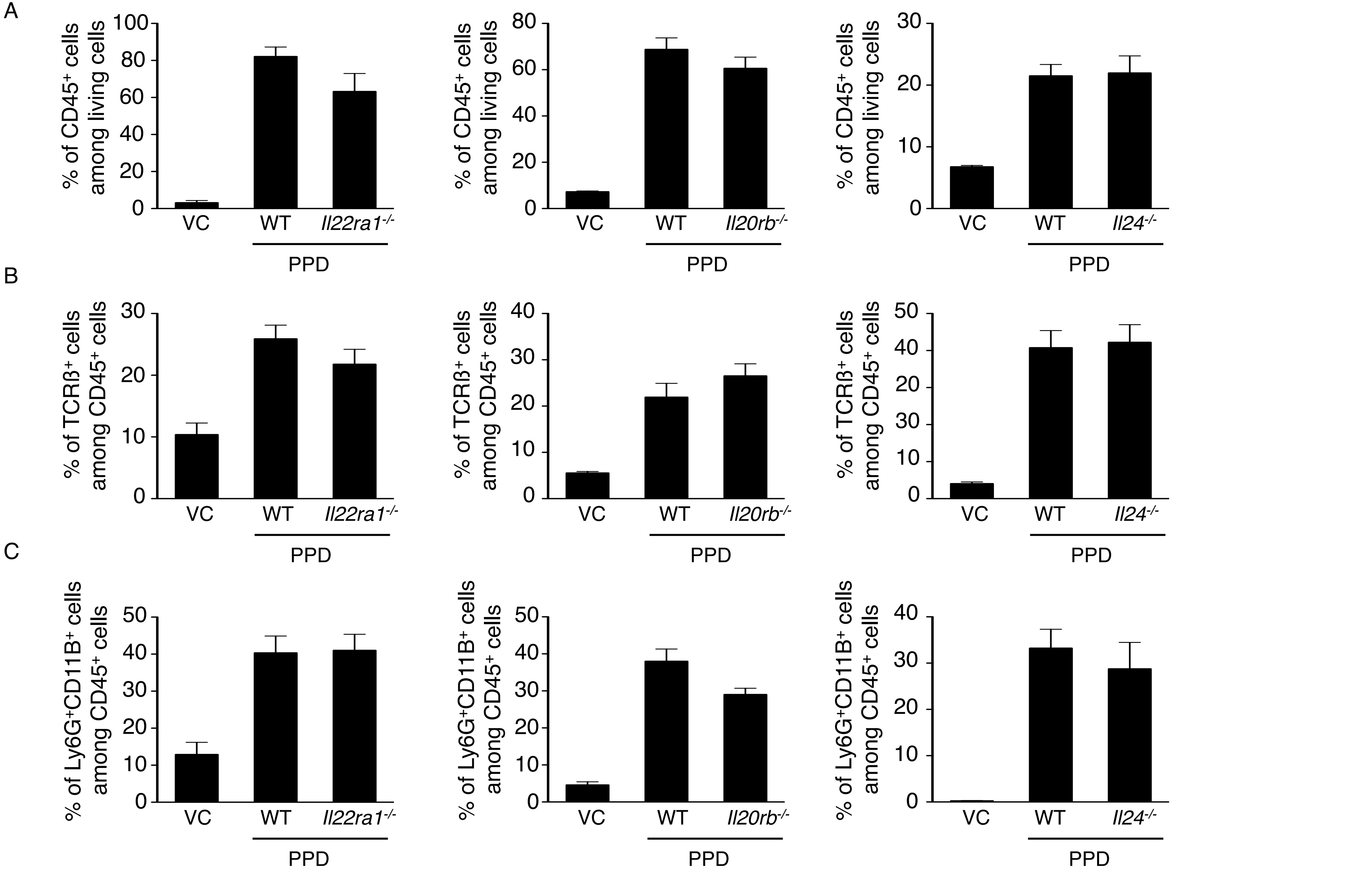

Suppl Figure 4: Inflammatory infiltrate is similar in the epidermis of WT and *Il22ra1$^{-/-}$*, *Il20rb$^{-/-}$* and *Il24$^{-/-}$* mice during late phase. Flow cytometry on epidermal cells from VC and PPD-treated mice. The skin was harvested 24 hours after the third PPD application. For CD45 (A), cells were gated on living cells and for TCRß (B) and Ly6G CD11B (C) cells were gated on CD45^{+} living cells. Data are means ± SEM (N = at least 6 mice per group). **$p < 0.01$ (Mann-Whytney to compare the two groups of treated mice). (VC = vehicle control).

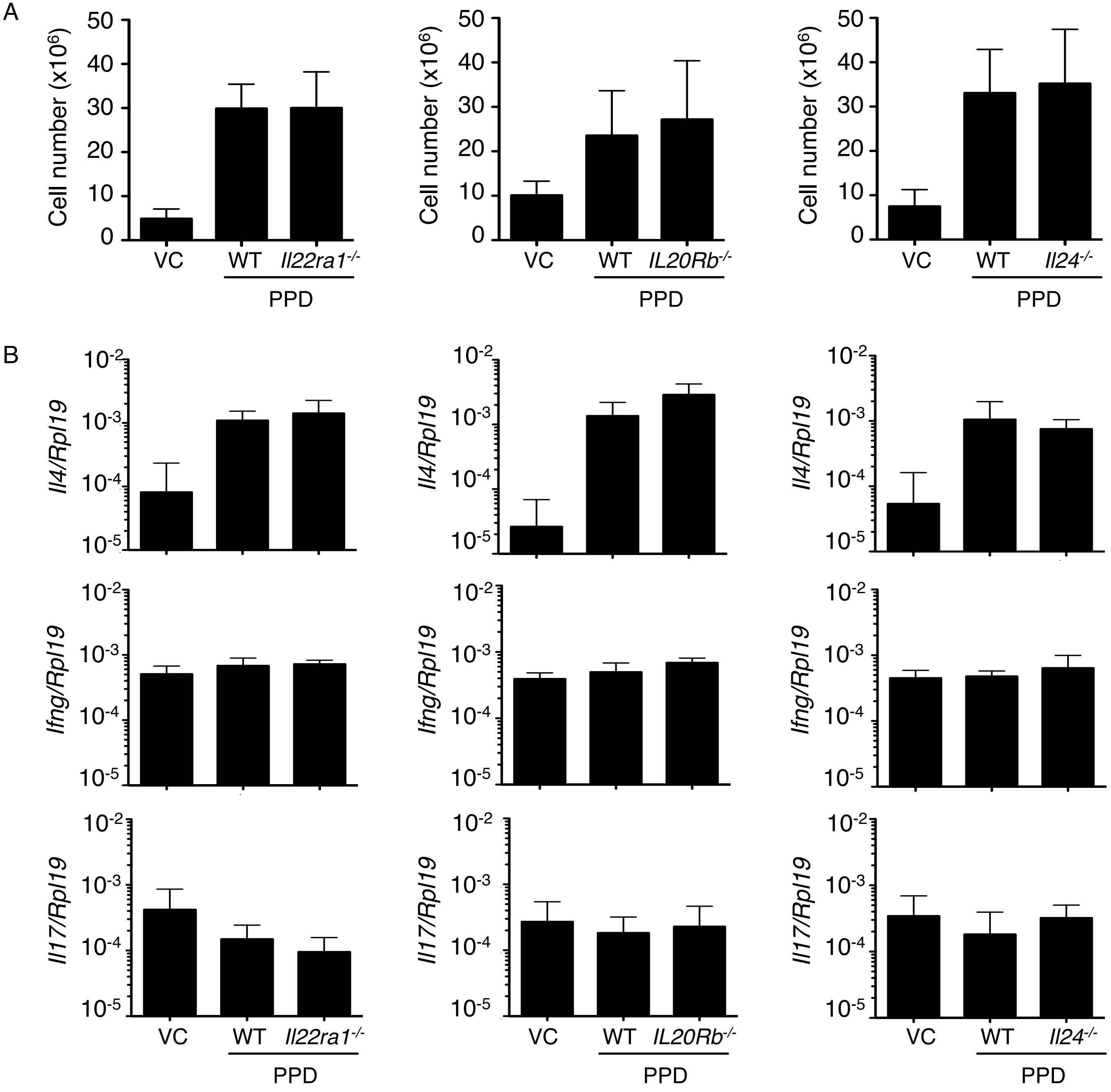

Suppl Figure 5: Cell numbers and cytokine expression are similar in auricular lymph nodes of WT and *Il22ra1*$^{-/-}$, *Il20rb*$^{-/-}$ and *Il24*$^{-/-}$ mice during late phase. A, Cell number was evaluated by counting auricular lymph node cells with Burker's chamber 24 hours after the third PPD application in VC and PPD-treated mice. B, RNA was isolated from auricular lymph node cells from VC and PPD-treated mice 24 hours after the third PPD application and quantitative RT-PCR analysis was performed for *Il4*, *Ifng* and *Il17*. Data are means ± SD (n = at least 4 mice per group, Mann-Whytney to compare the two groups of treated mice, no statistical difference). (VC = vehicle control).

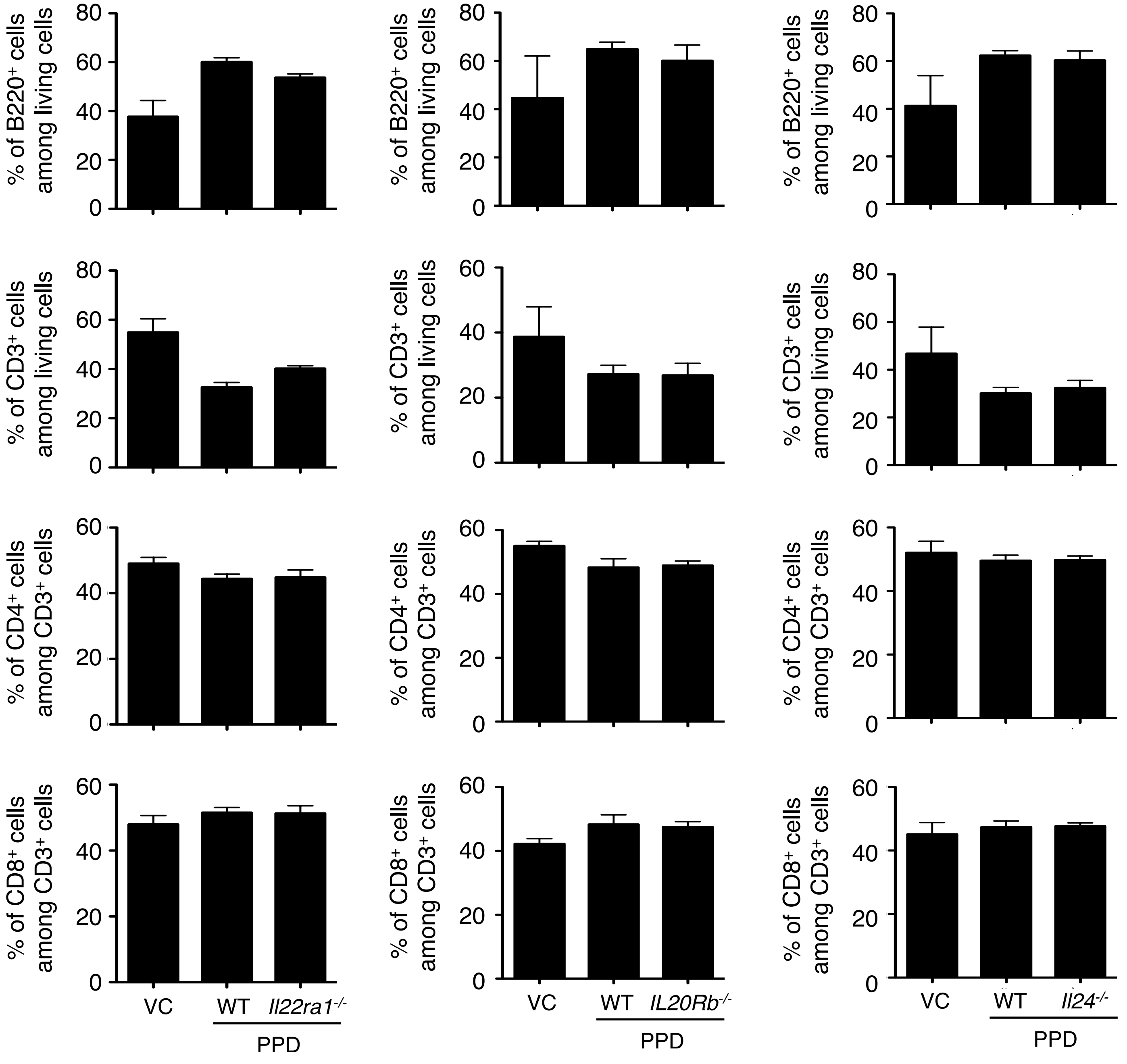

Suppl Figure 6: Cell composition is similar in auricular lymph nodes of WT and *Il22ra1*$^{-/-}$, *Il20rb*$^{-/-}$ and *Il24*$^{-/-}$ mice during late phase. 24 hours after the third PPD application, flow cytometry analysis was performed on auricular lymph node cells from VC and PPD-treated mice. For B220 and CD3, cells were gated on living cells and for CD4 and CD8, cells were gated on $CD3^+$ living cells. Data are means ± SD (n = at least 4 mice per group, Mann-Whytney to compare the two groups of treated mice, no statistical difference). (VC = vehicle control).

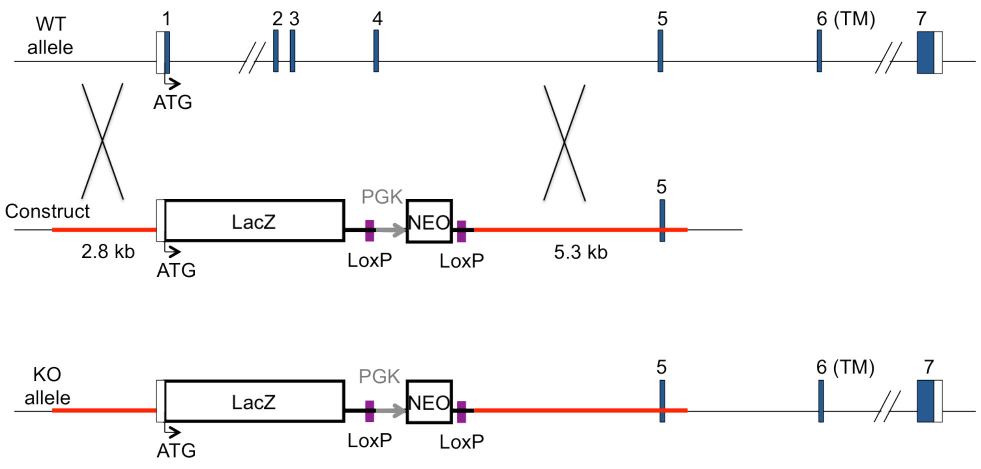

Suppl. Figure 7: The structure of the *Il22ra1* locus, the targeting vector, and the predicted homologous recombination (red) are shown. *Il22ra1*$^{-/-}$ mice were generated by targeting exons 1-4 in the R1 line (129X1/SvJ x 129S1) ES cells and backcrossed with 129sv mice for 15 generations. The targeting vector was constructed to replace the first four exons of the *Il22ra1* gene by a LacZ reporter cassette with a nuclear localization signal (pSKT NLS LacZ) in frame with initiation codon of *Il22ra1* and a neomycin-resistant cassette (PGK Neo A), which is floxed. The 5' arm of this construct consisted of a 2.8 kb fragment starting at a NheI site from the promoter and ending at the ATG initiation codon, and was inserted in frame with the LacZ cassette. The 3' arm consisted of a 5.3 kb fragment that extend from the XmaI restriction site, located between exon 4 and exon 5, and the BglII site located between exon 5 and exon 6.

The exons are shown as boxes, with white and blue boxes for non-coding and coding regions, respectively. The size of the 5' and 3' arms is indicated. Neo, neomycin-resistance cassette.

Patient	Age	Sensitization	Clinical reaction	Patch test results (ICDRG criteria)		
				8 hours	24 hours	48 hours
**1**	30	Hair dyes	Acute contact dermatitis scalp and face	-	++	+++
**2**	77	?	None	-	-	++
**3**	45	Hair dyes	Acute contact dermatitis scalp, face and neck	-	+++	+++
**4**	51	?	None	-	-	++
**5**	35	Hair dyes	Acute contact dermatitis face	-	++	++
**6**	50	Hair dyes	Chronic eczema neck	-	-	-
**7**	64	Hair dyes	?	-	++	+++
**8**	56	Hair dyes	Acute contact dermatitis face	-	++	+++
**9**	65	?	Armpit eczema	-	+	++
**10**	53	Hair dyes	Eyelid eczema	-	++	+++
**11**	52	Hair dyes	Acute contact dermatitis scalp	-	++	+++

Suppl. Table 1. Patient data and patch test results

Gene	Forward primer	Reverse primer	Taqman probe
mβ-actin	5’-CTCTGGCTCCTAGCACCATGAAG-3’	5’-GCTGGAAGGTGGACAGTGAG-3’	5’-TCGGTGGCTCCATCCTGGC-3’
mIfng	5’-TCAAGTGGCATAGATGTGGAAGAA-3’	5’-TGGCTCTGCAGGATTTTCATG-3’	5’-TCACCATCCTTTTGCCAGTTCCTCCAG-3’
mIl4	5’-GAACGAGGTCACAGGAGAAGG-3’	5’-GGACTCATTCATGGTGCAGCTTA-3’	5’-CCTCACAGCAACGAAGAACACCACAG-3’
mIl17	5’-GCTCCAGAAGGCCCTCAG-3’	5’-CTTTCCCTCCGCATTGACA-3’	5’-ACCTCAACCGTTCCACGTCACCCTG-3’
mIl19	5’- GAGCGATGTCAGGTGCACAGAC -3’	5’- CTTAAGGGCAGCAGATGAGACCTC -3’	5’- CTGCAGTCAGGAAGCCACCAATG -3’
mIl22	5’CGCTGCCCGTCAACACCCGG-3’	5’-CTGATCTTTAGCACTGACTCCTCG-3’	5’-TGAGGTGTCCAACTTCCAGCAGCCG-3’
mIl24	5’- CTACAGCCCAGTAAGGACAATTCC-3’	5’- GTCCACTTCCCCAAAGGCTTTCAC -3’	5’- CGCTGGTGTGCACTCTCACTAATGGG -3’
hEF1	5’- GCTTCACTGCTCAGGTGAT -3’	5’- GCCGTGTGGCAATCCAAT -3’	5’- AAATAAGCGCCGGCTATGCCCCTG -3’
hIL20	5’- GCCAATTCCTTTCTTACCATCAA -3’	5’- CCCACAATGGCATGTCATGT -3’	5’- AGGACCTCCGGCTCAGTCATGCC -3’
hIL22	5’- GCAGGCTTGACAAGTCCAACT -3’	5’- GCCTCCTTAGCCAGCATGAA -3’	5’- CCAGCAGCCCTATATCACCAACCGC -3’
hIL24	5’- GGCCCAGGGCCAAGAA -3’	5’- GATGTTATCCTGAGCTTGCATAGTGT -3’	5’- TGGGAAGCCTTCTGGGCTGTGAAA -3’
mRpl19	5’- CCTTGTCTGCCTTCAGCTTGT -3’	5’- GAAGGTCAAAGGGAATGTGTTCA -3’	
mIl20	5’- CATCAAGAAGGACCTCTCAGTCTG -3’	5’- CATCTCCTCCATCCATCTCAGAAG -3’	
mCD3e	5’- GGATGCGGTGGAACACTTTCTG -3’	5’- GTCAACTCTACACTGGTTCCTGAG -3’	
mKrt10	5’- CAAGTCTTCCGGTGGCGGCGAC -3’	5’- GGACTCTACCCTCAGGTGTCACCTC -3’	

Suppl. table 2 : Sequences of primers and probes used for quantitative RT-PCR
